# Public priorities for osteoporosis and fracture research: results from a general population survey

**DOI:** 10.1007/s11657-017-0340-5

**Published:** 2017-04-28

**Authors:** Zoe Paskins, Clare Jinks, Waheed Mahmood, Prakash Jayakumar, Caroline B. Sangan, John Belcher, Stephen Gwilym

**Affiliations:** 1 Arthritis Research UK Primary Care Centre, Research Institute for Primary Care Sciences, Keele University, Keele, Staffordshire ST5 5BG UK; 2 Haywood Academic Rheumatology Centre, Staffordshire and Stoke-on-Trent Partnership Trust, Stoke-on-Trent, ST6 7AG UK; 3 Oxford Trauma, Nuffield Department of Orthopaedics, Rheumatology and Musculoskeletal Sciences, University of Oxford, Oxford, UK; 4 National Osteoporosis Society, Bath, BA2 0PJ UK

**Keywords:** Research priorities, Patient and public involvement and engagement, Survey, Osteoporosis, Fracture

## Abstract

***Summary*:**

This is the first national study of public and patient research priorities in osteoporosis and fracture. We have identified new research areas of importance to members of the public, particularly ‘access to information from health professionals’. The findings are being incorporated into the research strategy of the National Osteoporosis Society.

**Purpose:**

This study aimed to prioritise, with patients and public members, research topics for the osteoporosis research agenda.

**Methods:**

An e-survey to identify topics for research was co-designed with patient representatives. A link to the e-survey was disseminated to supporters of the UK National Osteoporosis Society (NOS) in a monthly e-newsletter. Responders were asked to indicate their top priority for research across four topics (understanding and preventing osteoporosis, living with osteoporosis, treating osteoporosis and treating fractures) and their top three items within each topic. Descriptive statistics were used to describe demographics and item ranking. A latent class analysis was applied to identify a substantive number of clusters with different combinations of binary responses.

**Results:**

One thousand one hundred eighty-eight (7.4%) respondents completed the e-survey. The top three items overall were ‘Having easy access to advice and information from health professionals’ (63.8%), ‘Understanding further the safety and benefit of osteoporosis drug treatments’ (49.9%) and ‘Identifying the condition early by screening’ (49.2%). Latent class analysis revealed distinct clusters of responses within each topic including primary care management and self-management. Those without a history of prior fracture or aged under 70 were more likely to rate items within the cluster of self-management as important (21.0 vs 12.9 and 19.8 vs 13.3%, respectively).

**Conclusion:**

This is the first study of public research priorities in osteoporosis and has identified new research areas of importance to members of the public including access to information. The findings are being incorporated into the research strategy of the National Osteoporosis Society.

**Electronic supplementary material:**

The online version of this article (doi:10.1007/s11657-017-0340-5) contains supplementary material, which is available to authorized users.

## Introduction

Sixteen years ago, Tallon et al. published a high profile call to bridge the gap between research agendas and consumer views in osteoarthritis [1]. Patients wanted more rigorous evaluation of the effects of surgery and physiotherapy and better assessment of the educational and coping strategies that might help patients to manage this chronic, disabling and often painful condition. They had little enthusiasm for drug trials, yet these constituted the vast majority of the published studies of treatments for this condition [1, 2].

Historically, members of the public have been less likely to be consulted about research priorities than clinicians and other stakeholders [3]. A number of cultural assumptions have been identified which may underpin a reluctance to set priorities with patients. These include the belief that patients may shift focus away from basic science and a feeling that researchers are best placed to identify priorities [4].

Increasingly, policy makers and public funders recognise the importance of involving public and patients in setting priorities for research to ensure that research agendas are patient-centred, relevant and that research outcomes have a high likelihood of resulting in patient benefit. Ensuring that research addresses the priorities of research users is also one of four strategies proposed to reduce waste and increase value of research [5]. Over the last decade, a number of initiatives such as INVOLVE, part of the National Institute for Health Research (NIHR), have been established to facilitate and promote active public involvement in all aspects of research, including priority setting. The James Lind Alliance (JLA) was formed in 2004 and aimed to bring patients and clinicians together in a new way to identify and address important uncertainties about the effects of care and treatments [6]. These initiatives are by no means limited to the UK; existing literature evidences health research priority setting exercises with users in the USA, Canada and Australia in addition to the UK [7]. Furthermore, the European League Against Rheumatism (EULAR) has recognised the pivotal role of patients in the development of recommendations [8]. Unfortunately, despite this apparent revolution in patient engagement, recent evidence suggests the mismatch between the research patients want and the research that is conducted still persists [9]. Furthermore, a previous report commissioned by the JLA established that the majority of charitable funders in the UK funded research in a responsive mode, with only a minority funding research that only met pre-identified priorities [4]. With respect to osteoporosis, no studies to date have investigated the research priorities of patients and members of the public, which is surprising given the extent and impact of the condition [10, 11].

This study, conducted by researchers in conjunction with a charitable funder, the National Osteoporosis Society (NOS) aimed to prioritise, with patients and public members, a list of research topics for the osteoporosis research agenda.

## Methods

An e-survey to prioritise topics for research was co-designed with a patient research user group based on the findings from previous qualitative research using a combination of face-to-face methods to identify topics and online survey for voting is part of a suite of recommended methods by the Cochrane Methods Priority Setting group [12]. The categories and items for the e-survey were previously derived from four focus groups with members of the public conducted in two UK cities [13]. Participants for the focus groups were recruited from members of the National Osteoporosis Society (NOS) (Staffordshire) and a research cohort (Oxford) of individuals who had experienced fracture. In the focus groups, participants were asked to describe their experience of osteoporosis, what was important to them, what problems they had had, what was missing from their care/management and what could be improved. Qualitative analysis identified four topics (understanding and preventing osteoporosis, living with osteoporosis, treating osteoporosis and treating fractures). For each topic, a number of subthemes were identified (Table 1). Researchers worked with a patient research user group at Keele [14] to translate each subtheme into a questionnaire stem for the e-survey; ZP wrote an initial draft which the patient group subsequently edited, ensuring that each stem represented discussion in the focus group and was written in an understandable way [15]. The user group consisted of five existing members with long-term musculoskeletal conditions, three of whom had experience of advising a previous study regarding research priorities and three of whom had experience of osteoporosis and/or fracture (either personal experience or as a relative/spouse or carer).

**Table 1:** Topics and subthemes used for e-survey

Understanding and preventing osteoporosis (OP)	Living with osteoporosis	Treating osteoporosis	Treating fractures
Awareness of OP in general population Promoting bone health in • Schools • Well man/woman clinics Role of diet Role of exercise Identifying new causes Role of screening Understanding bone physiology Prognosis Genetics	Impact on • Activities of daily living • Employment • Relationships Managing pain Anxiety and depression Managing OP with comorbidity Fear of fracture Support from other organisations Role of advice and information Improving attitudes to OP	GP care Communication between primary and secondary care Self-management Safety and benefit of supplements Safety and benefit of drugs Complementary treatments Role of exercise Stratified treatment Follow up: role of • DXA • Annual review	Diagnosis of fractures Managing pain Short-term occupational therapy Period of immobilisation Effect of OP and drugs on fracture healing Role of exercise Speed of recovery Short–medium-term rehabilitation Wound management Long-term risk/benefit of surgery

The resultant survey had four sections (representing the four topics), each containing 10 stems. Responders were asked to indicate their top three items (stems) within each topic. A fifth question asked respondents to indicate their most important topic out of the four sections. Finally, participants were asked about their age, gender, self-reported diagnosis of osteoporosis and fracture history. The full e-survey is available in Supplementary Data.

The e-survey was built within a freely available Web tool (Survey Monkey) and a URL link to the e-survey was disseminated to approximately 16,000 supporters of the UK National Osteoporosis Society (NOS) in one monthly e-newsletter, in December 2015. The e-survey was also advertised via social media, via a page on the society’s website and the society’s quarterly membership magazine.

The e-survey remained open for 1 month. After this period, responses were exported into Excel. Descriptive statistics were used to describe demographics of responders and the top three items within each topic. In order to identify the top 10 priorities overall, a weighting was necessary. Participants had to choose three items within each question, therein simply showing the count of responses for each item would not reflect what the participants felt was most important across all four categories. The responses to all 40 stems were pooled, with each individual’s three items in their most important topic (as indicated by question 5) receiving a double weighting.

A latent class analysis was applied to identify a substantive number of clusters with different combinations of binary responses. Latent class analysis (LCA) is a statistical method for finding subtypes of related cases (latent classes) from multivariate categorical data. For example, it can be used to find distinct diagnostic categories given presence/absence of several symptoms, types of attitude structures from survey responses, consumer segments from demographic and preference variables, or examinee subpopulations from their answers to test items. The results of LCA can also be used to classify cases to their most likely latent class. The data analysis for this paper was generated using SAS software 9.3[Copyright © 2002–2010 by SAS Institute Inc., Cary, NC, USA]. The optimum number of classes was determined by a combination of the following: (1) goodness-of-fit statistics (Akaike Information Criteria, Bayesian Information Criteria (BIC), sample size-adjusted BIC, and the Lo-Mendell-Rubin adjusted likelihood ratio test (LRT)); (2) uncertainty of classification measures such as the entropy and average posterior probabilities; (3) class size of at least 10% of the sample and (4) clinical relevance and interpretability. Labels for each cluster were determined by consensus between four authors (CJ, ZP, PJ, SG). Where the contingency table is larger than 2 × 2, Fisher-Freeman-Halton exact test of independence was used to quantify the association between responses within any cluster and age, fracture, diagnosis of osteoporosis, and gender.

## Results

### Characteristics of responders

One thousand one hundred eighty-eight respondents (approximately 7.4%) completed the e-survey. Of the responders, 87.4% were female, 295 (24.8%) aged under 60, 537 (45.2%) aged 60–69 and 356 (30%) aged 70 or over. The majority reported a diagnosis of osteoporosis (79%) and 39.6% reported a history of fracture. Those not reporting a history of osteoporosis or fracture were assumed to have an interest in the condition by virtue of their interest in the NOS; this group is likely to represent individuals who consider themselves at risk of osteoporosis in addition to family members, carers or possibly health care professionals. Interestingly, 10.3% reported being unsure as to their fracture history. The NOS were not able to release information about the gender or age of their membership/mailing list due to data protection reasons, and therefore, it is not possible to draw any conclusions about the difference between responders and non-responders. Characteristics of responders are further detailed in Table 2.

**Table 2:** Characteristics of e-survey responders

	Number (%)
Female Male Missing	1038 (87.4) 88 (7.4) 62 (5.2)
Age	
under 50 50–59 60–69 70–79 80 and over Not recorded	95 (8.0) 200 (16.8) 537 (45.2) 250 (21.0) 50 (4.2) 56 (4.7)
Self-reported diagnosis of osteoporosis	
Yes No Unsure Missing	939 (79.0) 176 (14.8) 12 (1.0) 61 (5.1)
Self-reported history of fracture	
Yes Fracture site^a^ Radial Hip Vertebral Pelvis Humerus Other Number of fracture sites 1 fracture site 2 fracture sites ≥3 fracture sites No Unsure Missing	471 (39.6) 149 37 227 14 48 176 322 103 45 547 (46.0) 122 (10.3) 48 (4.0)

^a^Participants could select multiple responses; hence, total does not equal 471

### Highest scoring items in weighted analysis: top ten

The most important topic was rated as ‘understanding and preventing osteoporosis’ (*n* = 470, 39.6%); followed by ‘treating osteoporosis’ (*n* = 373, 31.4%); ‘living with osteoporosis’ (*n* = 255, 21.5%) and ‘treating fractures’ (*n* = 51, 4.3%). Thirty-nine respondents did not answer this question and these were not included in the weighted analysis. The top scoring three items within each topic are detailed in Supplementary data Table S1. The top three items overall were ‘Having easy access to advice and information from health professionals’, ‘Understanding further the safety and benefit of osteoporosis drug treatments’ and ‘Identifying the condition early by screening’. The top ten items determined by the weighted analysis are shown in Table 3.

**Table 3:** Top 10 items overall

	Weighted score^a^	Number (%) of participants who rated as important
1. Having easy access to advice and information from health professionals	872	758 (63.8)
2. Identifying the condition early by screening	798	585 (49.2)
3. Understanding further the safety and benefit of osteoporosis drug treatment	797	593 (49.9)
4. The impact of osteoporosis on being able to do daily activities	674	543 (45.7)
5. The effect of osteoporosis and osteoporosis drugs on fracture healing	656	640 (53.9)
6. Improving confidence to reduce fear of fracture	627	539 (45.4)
7. Pain associated with the condition	619	497 (41.8)
8. Identifying which types of exercise are best after fracture	617	604 (50.8)
9. Understanding further the role of diet in keeping bones healthy	577	405 (34.1)
10. Managing osteoporosis when you have other ongoing health conditions	570	467 (39.3)

^**a**^Derived by pooling responses to all 40 stems, with each individual’s three items in their most important topic (as indicated by question 5) receiving a double weighting. Range of weighted scores = 25–872; median = 442

### Latent class analysis

Using LCA within each topic, distinct clusters of responses to related items were identified, as described in Table 4. Although we aimed for a class size of at least 10% of the sample, clinical interpretation of the third topic, ‘treating osteoporosis’, revealed six to be the optimum number of clusters, despite one cluster representing only 8.8% of the sample. The summary of latent class diagnostics are detailed in Supplementary Tables S2– S5.

**Table 4:** Clusters (classes) of items within each topic derived from latent class analysis

	Understanding and preventing osteoporosis (OP)		Living with osteoporosis		Treating osteoporosis		Treating fractures	
Label and number of respondents (%) for each class^a^ (most frequently occurring cluster in italics)	C1 *Promoting early diagnosis*	438 (36.9)	C1 Support and information	229 (19.3)	C1 *Primary care management*	240 (20.2)	C1 Acute fracture care (diagnosis and management)	415 (34.9)
	C2 Self-management	199 (16.8)	C2 *Managing the effects of the condition*	429 (36.1)	C2 Personalised medicine	104 (8.8)	C2 *Medium to long-term impact of fracture management*	445 (37.5)
	C3 Increasing scientific knowledge	323 (27.2)	C3 Managing fear of fracture	283 (23.8)	C3 Monitoring the condition	228(19.2)	C3 Optimising return to function	328 (27.6)
	C4 Understanding causes	228 (19.2)	C4 Managing pain	247 (20.8)	C4 Self-management	225 (18.9)		
					C5 Monitoring drug effectiveness	161 (13.6)		
					C6 Safety and benefit of medication and supplements	229 (19.3)		

^a^A class describes a group of items within a topic. The label describes how the items link together. Most frequently occurring cluster highlighted in italics

**Fig. 1:**
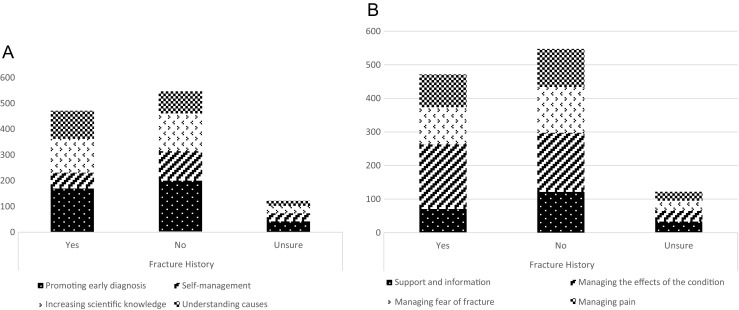
Number of patients in each cluster with and without fracture: questions 1 and 2

Responses in questions 1 and 2 (understanding and preventing osteoporosis, and living with osteoporosis) were associated with self-reported history of fracture (*p* < 0.001, *p* = 0.03) (Fig. 1), but not with age or gender. Responses in questions 1 (understanding and preventing osteoporosis), but not question 2 (living with osteoporosis) were associated with self-reported history of diagnosis of osteoporosis (*p* < 0.001). Responses in questions 3 and 4 (treating osteoporosis and treating fractures) were associated with age (*p* = 0.048, *p* = 0.03), but not with self-reported history of fracture, diagnosis of osteoporosis or gender.

**Fig. 2:**
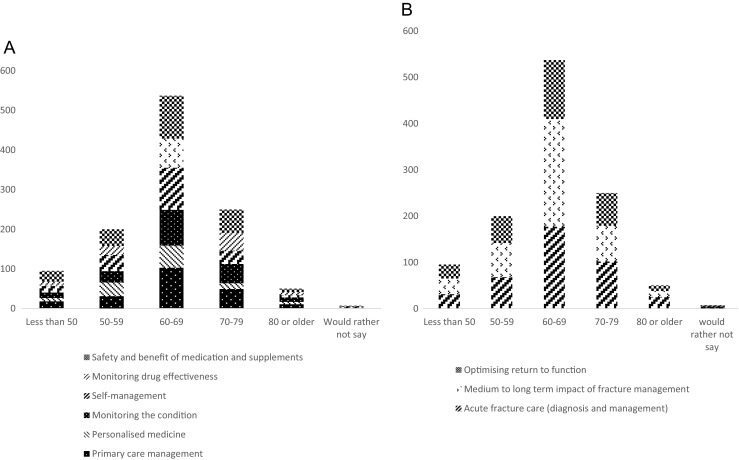
Number of patients in each cluster by age: questions 3 and 4

Those with prior fracture had a higher probability than those without prior fracture of rating items within ‘understanding causes’ (21.1 vs 15.7%, Fig. 1a) and ‘managing the effects of the condition’ (41 vs 32.3%, Fig. 1b) as important. Those with a self-reported diagnosis of osteoporosis had a lower probability than those without diagnosis of rating items in the cluster ‘promoting early diagnosis’ (33.9 vs 48.9%) as important and a higher probability of rating items in the cluster ‘understanding causes’ (20.0 vs 10.2%) as important. Those without prior fracture had a higher probability than those with prior fracture of rating items in the cluster ‘self-management’ (21.0 vs 12.9%, Fig. 1a) and support and information (22.1 vs 14.9%, Fig. 1b) as important. Those aged 70 and over had a higher probability than those aged under 70 of rating items in the cluster ‘monitoring drug effectiveness’ (17.7 vs 12.9%, Fig. 2a) and acute fracture care (42.3 vs 33.2%, Fig. 2b) as important. Those aged under 70 had a higher probability than those aged 70 and over of rating items within the clusters ‘self-management’ (19.8 vs 13.3%, Fig. 2a) and ‘medium to long term impact of fracture’ as important (41.1% vs 30.0%, Fig. 2b).

## Discussion

This study reports for the first time, topics of importance to public and patients in the research of osteoporosis and fracture. Currently, the existing published research priorities for osteoporosis are detailed only in national guidance documents. Published research recommendations largely focus on safety, benefit and optimal duration of drug treatment, algorithms for screening and fracture risk assessment and further epidemiology to understand causes of osteoporosis [16–18]; the SIGN guidance in the UK included recommendations for research relating to nutrition and optimal frequency of DXA scanning and the National Osteoporosis Federation include research questions relating to exercise treatments, assessing bone strength, assessing and reducing falls and diagnosing vertebral fractures [19, 20]. Some national guidance documents do not contain any specific research recommendations [21, 22].

This work emphasises that patients and public members would value further health services research into information giving, primary care management and non-pharmacological management in addition to studies on safety and benefit of drugs, and studies that explore outcomes other than fracture. Within the top three, screening and safety and benefit of drugs are aligned with established research recommendations. However, the number one area of importance, having easy access to information from health professionals, is not represented in guideline research recommendations. This finding is concordant with priority setting in other long-term conditions, both musculoskeletal and others, where education and communication are highly rated [2, 23, 24].

The top ten features non-drug aspects of management such as exercise and diet; again, this is in line with findings from a review of JLA priority setting partnerships in 14 long-term conditions where only 18% of the top ten research uncertainties relating to treatment and concerned drugs [9]. This is important because there is a significant mismatch between the volume of ongoing trials relating to drug treatment and patient priorities [9]. Research into self-management has been rated an important priority by patients in priority setting exercises in joint pain and other long-term conditions such as asthma [25, 26]. The top ten list also highlights the important outcomes to patients with evidence that pain, fear of fracture and difficulty with activities of daily living need to be prioritised in future research, in addition to fracture prevention.

By drawing together clusters of similar items which did not score so highly individually, the latent class analysis findings are useful to further illustrate areas of priority. Of the most frequent clusters, primary care management stands out as a topic of importance that is not represented in the top ten, highlighting the importance of health services research in this area. Specific survey items within the primary care cluster included the evaluation of annual review clinics for patients with osteoporosis and improving care and support from patients’ general practitioners (family physicians). ‘Increasing scientific knowledge’ is a further area of importance identified in the latent class analysis, which is of interest given that there is a perception that patients will not prioritise basic science research uncertainties, although other work with patients has demonstrated that patients do value biomedical research [27]. The Fischer’s exact testing demonstrated that individuals with self-reported diagnoses of fracture and osteoporosis were more likely to rate items related to ‘understanding causes’ as important which is perhaps not a finding of surprise. Older individuals were more likely than younger individuals to rate research into drugs, and less likely to rate self-management as important. This is in keeping with findings from a study of patients with joint pain which found older patients were more likely to consider medical interventions important [26].

Focusing on the views of patients and public alone, in contrast to other popular priority setting methods where clinicians are also involved, ensures health professionals do not influence patient and caregiver responses. However, our study is subject to some limitations. Although the response rate was less than 10%, the total number of respondents numbered over a thousand, and was in keeping with [28], or in excess of, the number of respondents to similar e-surveys in other national priority setting exercises [29]. The survey was predominantly available electronically and so may not have included the views of those without access to the Internet. Paper surveys were available, although none were requested. Government statistics in the UK suggest that Internet use in the elderly is on the increase with four in ten adults over 75 and 74% adults aged 65–75 accessing the Internet [30]. However, in view of the fact that the prevalence of osteoporotic fractures in general (particularly hip and vertebral) increases with age, our age distribution may not have been entirely representative. The survey may not have represented some minority groups; we did not collect ethnicity data. Similarly, although men are less likely to have osteoporosis than women, proportionately they may have been under-represented in our responders. However, in the latent class analysis, those men who did respond did not differ in their priorities to women.

Criticisms of the priority setting approaches in general include the lack of specificity of the resultant top ten items/research questions [31]. Unlike clinicians and researchers, patients think in terms of broad themes and quality of life outcomes rather than specific questions and interventions [32]. Translating research priority lists into commissioning briefs for funders is essential to make these priorities meaningful. Techniques for this include further work with patients and public to scope out the details of the topics and developing research recommendations using the EPICOT format (evidence, population, intervention, comparator, outcome and time period) [4]. Further work to ‘evidence map’ the existing knowledge, to compare with priorities and identify gaps would also be useful [33].

## Conclusion

This study reports for the first time, the research priorities of patients and public with respect to osteoporosis and fracture identified through a national e-survey. The findings have added to existing recommendation by identifying new topics, particularly, highlighting that ‘easy access to information from health professionals’ is of the highest importance to patients. Our partnership with the NOS will ensure that these priorities are positively realised and not resigned to history as another top ten. The findings from this research will be incorporated into the new research strategy for the charity, which will outline the journey the NOS research programme plans to embark on in order to bring about real improvements to the lives of people affected by osteoporosis.

## Electronic supplementary material

ESM 1 (DOCX 94 kb)

Table S1 (DOCX 17 kb)

Table S2 (DOCX 17 kb)

Table S3 (DOCX 17 kb)

Table S4 (DOCX 17 kb)

Table S5 (DOCX 17 kb)
