# Supplementary material for: Public priorities for osteoporosis and fracture research: results from a general population survey
Source: Arch Osteoporos. 2017 Apr 28;12(1):45. doi: 10.1007/s11657-017-0340-5 (PMC5409917; doi:10.1007/s11657-017-0340-5)
Supplement: Supplementary file 1 — (DOCX 94 kb) [file 11657_2017_340_MOESM1_ESM.docx]

**Your views on research in osteoporosis**

| We are conducting an e-survey about public views on research in osteoporosis. The e-survey has been designed by researchers at Keele University working in collaboration with members of the public with an interest in osteoporosis. We are seeking your views about what type of research you think we should be doing in osteoporosis. You do not need to know anything about research to take part in this study. You just need to be interested in osteoporosis.  You might find it helpful to think about   - what services are missing - what could be improved, or - what is important to you   The results of this survey will inform the National Osteoporosis Society’s research strategy and will be used to help decide how to give funding for future osteoporosis research.  The questionnaire should take less than **10 minutes** to complete.  All answers you provide will be anonymised and the data will be handled and stored confidentially according to the University’s research code of practice. Completing and submitting this questionnaire is taken as consent to taking part in the study.  If you have any questions about this questionnaire or the study in general please contact Zoe Paskins on 01782 733975 or Waheed Mahmood on [w.mahmood@keele.ac.uk](mailto:w.mahmood@keele.ac.uk)  Thank you for reading this information. |
| --- |

| **Instructions for completing the questionnaire**  Please answer questions by ticking the appropriate boxes.  There are no ‘correct’ or ‘incorrect’ answers. |
| --- |

**Thank you for your help with this study**

**Section 1: Your views about research**

| \| **Question 1. Understanding and preventing osteoporosis**  This question is about which topics should be researched to help us **know more about osteoporosis and to help prevent it.**  Please look at the list of topics below and select **THREE** which are the **most important** to you \| \| \| --- \| --- \| \| 1. Increasing awareness in the general population of what osteoporosis is \|  \| \| \| 1. Promoting the importance of maintaining healthy bones in schools \|  \| \| \| 1. Promoting the importance of maintaining healthy bones in well man or well woman clinics (e.g. in health checks) \|  \| \| \| 1. Understanding further the role of diet in keeping bones healthy \|  \| \| \| 1. Understanding further the role of exercise in keeping bones healthy \|  \| \| \| 1. Identifying new causes of osteoporosis e.g. health conditions or medicines \|  \| \| \| 1. Understanding the importance of genetics and/or having a family member with osteoporosis \|  \| \| \| 1. Identifying the condition early by screening \|  \| \| \| 1. Understanding further what happens inside the bone and the effect of treatment on the bone \|  \| \| \| 1. Understanding what the future holds after diagnosis \|  \| \|   **Question 2. Living with osteoporosis**  This question is about which topics should be researched to help people **live with osteoporosis.**  Please look at the list of topics below and select **THREE** which are the **most important** to you | |
| --- | --- | --- | --- | --- | --- | --- | --- | --- | --- | --- | --- | --- | --- | --- | --- | --- | --- | --- | --- | --- | --- | --- | --- | --- | --- | --- | --- | --- | --- | --- | --- | --- | --- |
| a. Having easy access to advice and information from health professionals |  |
| b. The impact of osteoporosis on being able to do daily activities |  |
| c. The impact of osteoporosis on employment |  |
| d. The impact of osteoporosis on relationships |  |
| e. Pain associated with the condition |  |
| f. Anxiety, depression and/or feeling low because of osteoporosis |  |
| g. Managing osteoporosis when you have other ongoing health conditions |  |
| h. Having easy access to help and support from other organisations |  |
| 1. Improving confidence to reduce fear of fracture |  |
| j. Improving the attitudes of others to the condition |  |
| **______________________________________________________________**   \| **Question 3. Treating osteoporosis**  This question is about which topics should be researched to **help treat people with osteoporosis.** Please look at the list of topics below and select **THREE** which are the most important to you \| \| \| --- \| --- \| \| a. Improving care and support from your GP \|  \| \|  \|  \|  \|  \|  \|  \| \| b. Improving partnership and communication between people with osteoporosis, their GPs and hospital services \|  \| \|  \|  \|  \|  \|  \|  \| \| c. Knowing more about what people can do themselves to manage their osteoporosis \|  \| \|  \|  \|  \|  \|  \|  \| \| d. Understanding further the safety and benefit of calcium and/ or vitamin D supplements \|  \| \|  \|  \|  \|  \|  \|  \| \| e. Understanding further the safety and benefit of osteoporosis drug treatments \|  \| \|  \|  \|  \|  \|  \|  \| \| f. Understanding further the safety and benefit of different exercises \|  \| \|  \|  \|  \|  \|  \|  \| \| g. Understanding further the safety and benefit of complementary/ alternative treatments or therapies \|  \| \|  \|  \|  \|  \|  \|  \| \| h. Understanding if there are different types of osteoporosis that should be treated differently \|  \| \|  \|  \|  \|  \|  \|  \| \| 1. To know how often to repeat bone density (DXA) scans for best care \|  \| \|  \|  \|  \|  \|  \|  \| \| j. Understanding the role and value of an annual review for osteoporosis e.g. with a practice nurse \|  \| \|  \|  \|  \|  \|  \|  \| \| **_________________________________________________________________** \| \|   **Question 4. Helping people with broken bones or fractures**  This question is about which topics should be researched to **help people with broken bones (or fractures).**  Please look at the list of topics below and select **THREE** which are the most important to you | |
| 1. Exploring better ways of diagnosing fractures |  |
| b. Managing pain resulting from a fracture |  |
| c. Having timely assessment and help with activities of daily living in the short term after fracture |  |
| d. Deciding how long a bone is immobilised in a cast or splint (rested) |  |
| e. The effect of osteoporosis and osteoporosis drugs on fracture healing |  |
| f. Identifying which types of exercise are best after fracture |  |
| g. Ensuring fastest return to work or leisure activities |  |
| h. Follow up for rehabilitation in the weeks after fracture |  |
| i. Wound management after surgery |  |
| j. Long term risks and benefits of fracture surgery (or metalwork) |  |

| **Question 5**  Thinking about **the questions you have just answered (Questions 1-4),** which **ONE area** is **most important to you?** | |
| --- | --- |
| Understanding more about and preventing osteoporosis (Q1) | **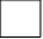** |
| Helping people live with osteoporosis, improving quality of life (Q2) | **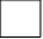** |
| Helping treat people with osteoporosis, improving services (Q3) | **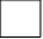** |
| The treatment of people with broken bones (fractures) (Q4) | **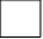** |
| **Question 6** |  |

Is there anything else you would like to tell us about what you think is important for research into osteoporosis?

|  |
| --- |

**Section 2: About you**

1. Have you been diagnosed with osteoporosis? (Please tick **one box** only).

Yes No Don’t know

Would rather not say

1. Are you Male Female
2. What is your age? (Please tick **one box** only).

Less than 50 50-59 60-69

70-79 80 or older would rather not say

1. Have you broken a bone (fracture) as a result of osteoporosis

Yes No

1. If you answered Yes to the previous question, please state what type of fracture (tick all that apply)

Hip (femur) Wrist vertebrae (spine)

Pelvis Arm (humerus) Other, please specify

…………………………………………………………………………………………………

…………………………………………………………………………………………………

I agree to submitting this survey and my anonymised information being used by the study team as described above.

Thank you for completing this survey

End
