# Supplementary material for: Public priorities for osteoporosis and fracture research: results from a general population survey
Source: Arch Osteoporos. 2017 Apr 28;12(1):45. doi: 10.1007/s11657-017-0340-5 (PMC5409917; doi:10.1007/s11657-017-0340-5)
Supplement: Supplementary file 2 — (DOCX 17 kb) [file 11657_2017_340_MOESM2_ESM.docx]

Supplementary data: Table 1: Highest rating topics and top three items within each topic

| **Topic and top 3 items within each topic** | **Number (%) rating**  **highest importance (topic)**  **and number (%) rating one of top 3 most important (items)** |
| --- | --- |
| 1. Understanding and preventing osteoporosis   1. Identifying the condition early by screening 2. Understanding further the role of diet in keeping bones healthy 3. Understanding further the role of exercise in keeping bones healthy | 470 (39.6)  585 (49.2)  405 (34.1)  376 (31.6) |
| 2. Treating osteoporosis   1. Understanding further the safety and benefit of osteoporosis drug treatments 2. Knowing more about what people can do themselves to manage their osteoporosis 3. To know how often to repeat bone density (DXA) scans for best care | 373 (31.4)  593 (49.9)  463 (39.0)  363 (30.6) |
| 3. Living with osteoporosis   1. Having easy access to advice and information from health professionals 2. The impact of osteoporosis on being able to do daily activities 3. Improving confidence to reduce fear of fracture | 255 (21.5)  758 (63.8)  543 (45.7)  539 (45.4) |
| 4. Treating fractures   1. The effect of osteoporosis and osteoporosis drugs on fracture healing 2. Identifying which types of exercise are best after fracture 3. Managing pain resulting from a fracture | 51 (4.3)  640 (53.9)  604 (50.8)  491 (41.3) |
