# Supplementary material for: Public priorities for osteoporosis and fracture research: results from a general population survey
Source: Arch Osteoporos. 2017 Apr 28;12(1):45. doi: 10.1007/s11657-017-0340-5 (PMC5409917; doi:10.1007/s11657-017-0340-5)
Supplement: Supplementary file 3 — (DOCX 17 kb) [file 11657_2017_340_MOESM3_ESM.docx]

| ***1 Class*** |  | ***2 Classes*** | ***3 Classes*** | ***4 Classes*** | ***5 Classes*** | ***6 Classes*** | ***7 Classes*** | ***8 Classes*** |
| --- | --- | --- | --- | --- | --- | --- | --- | --- |
| 3589.55  3640.35  3608.59  3650.35  1.00  na | *AIC*  *BIC*  *Sample Adj. BIC*  *Consistent AIC*  *Entropy*  *Bootstrapped*  *Likelihood Ratio*  *Test (SAS)* | 3148.05  3254.73  3188.02 3275.73  0.98  2 v 1  0.01 | 2972.08  3134.64  3033.00  3166.64  0.94  3 v 2  0.01 | 2882.20  3100.64 ᵻ  2964.06  3143.64 ᵻ  0.95  4 v 3  0.01 | 2833.70  3108.02  2936.50  3162.02  0.95  5 v 4  0.01 | 2812.11  3142.31  2935.84  3207.31  0.94  6 v 5  0.01 | 2771.93  3158.01  2916.61  3234.01  0.95  7 v 6  0.01 | 2743.26 ᵻ  3185.23  2908.88 ᵻ  3272.23  0.96  8 v 7  0.01 |
| C= | *% for each class* | C1= 0.6823  C2= 0.3177 | C1= 0.5592  C2= 0.1690  C3= 0.2718 | C1= 0.3692  C2= 0.1679  C3= 0.2715  C4= 0.1915 | C1= 0.2234  C2= 0.2173  C3= 0.1811  C4= 0.2515  C5= 0.1267 | C1= 0.2671  C2= 0.1311  C3= 0.1700  C4= 0.1499  C5= 0.1250  C6= 0.1570 | C1= 0.1632  C2= 0.2193  C3= 0.1075  C4= 0.0381  C5= 0.0720  C6= 0.2455  C7= 0.1545 | C1= 0.0701  C2= 0.2337  C3= 0.0709  C4= 0.1211  C5= 0.1051  C6= 0.0624  C7= 0.2158  C8= 0.1209 |

**Supplementary data Table 2: Summary of Latent Class Diagnostics for Question 1**

(ᵻ) Lowest Information Criteria (IC) value
