# Supplementary material for: Public priorities for osteoporosis and fracture research: results from a general population survey
Source: Arch Osteoporos. 2017 Apr 28;12(1):45. doi: 10.1007/s11657-017-0340-5 (PMC5409917; doi:10.1007/s11657-017-0340-5)
Supplement: Supplementary file 4 — (DOCX 17 kb) [file 11657_2017_340_MOESM4_ESM.docx]

| ***1 Class*** |  | ***2 Classes*** | ***3 Classes*** | ***4 Classes*** | ***5 Classes*** | ***6 Classes*** | ***7 Classes*** | ***8 Classes*** |
| --- | --- | --- | --- | --- | --- | --- | --- | --- |
| 3130.26  3181.06  3149.30  3191.06  1.00 | *AIC*  *BIC*  *Sample Adj. BIC*  *Consistent AIC*  *Entropy*  *Bootstrapped*  *Likelihood Ratio*  *Test (SAS)* | 2831.40  2938.09  2871.38  2959.09  0.98  2 v 1  0.01 | 2642.00  2804.56  2702.92  2836.56  0.99  3 v 2  0.01 | 2544.23  2762.68  2626.09  2805.68  0.99  4 v 3  0.01 | 2480.18  2754.50  2582.97  2808.50  0.99  5 v 4  0.01 | 2393.51  2723.72  2517.25  2788.72  0.99  6 v 5  0.01 | 2242.79  2628.87 ᵻ  2387.47  2704.87 ᵻ  0.99  7 v 6  0.01 | 2189.92 ᵻ  2631.88  2355.54 ᵻ  2718.88  0.99  8 v 7  0.01 |
| C= | *% for each class* | C1= 0.6380  C2= 0.3620 | C1= 0.3515  C2= 0.3613  C3= 0.2872 | C1= 0.1931  C2= 0.3613  C3= 0.2379  C4= 0.2077 | C1= 0.1929  C2= 0.3611  C3= 0.1500  C4= 0.1588  C5= 0.1372 | C1= 0.1925  C2= 0.3612  C3= 0.0488  C4= 0.1587  C5= 0.1375  C6= 0.1013 | C1= 0.1855  C2= 0.3600  C3= 0.0873  C4= 0.0702  C5= 0.0496  C6= 0.0883  C7= 0.1590 | C1= 0.0412  C2= 0.3569  C3= 0.0488  C4= 0.0793  C5= 0.0496  C6= 0.1103  C7= 0.1592  C8= 0.1547 |

**Supplementary data Table 3: Summary of Latent Class Diagnostics for Question 2**

(ᵻ) Lowest Information Criteria (IC) value
