# Supplementary material for: Public priorities for osteoporosis and fracture research: results from a general population survey
Source: Arch Osteoporos. 2017 Apr 28;12(1):45. doi: 10.1007/s11657-017-0340-5 (PMC5409917; doi:10.1007/s11657-017-0340-5)
Supplement: Supplementary file 5 — (DOCX 17 kb) [file 11657_2017_340_MOESM5_ESM.docx]

| ***1 Class*** |  | ***2 Classes*** | ***3 Classes*** | ***4 Classes*** | ***5 Classes*** | ***6 Classes*** | ***7 Classes*** | ***8 Classes*** |
| --- | --- | --- | --- | --- | --- | --- | --- | --- |
| 3260.30  3311.10  3279.34  3321.10  1.00 | *AIC*  *BIC*  *Sample Adj. BIC*  *Consistent AIC*  *Entropy*  *Bootstrapped*  *Likelihood Ratio*  *Test (SAS)* | 3090.89  3197.57  3130.87  3218.57  0.98  2 v 1  0.01 | 3043.43  3205.99  3104.34  3237.99  0.98  3 v 2  0.01 | 2918.15  3136.59  3000.00  3179.59 ᵻ  0.98  4 v 3  0.01 | 2855.11  3129.43  2957.91  3183.43  0.85  5 v 4  0.01 | 2792.54  3122.74 ᵻ  2916.28 ᵻ  3187.74  0.87  6 v 5  0.01 | 2794.29  3180.37  2938.97  3256.37  0.86  7 v 6  0.65 | 2772.66 ᵻ  3214.62  2938.28  3301.62  0.92  8 v 7 |
| C= | *% for each class* | C1=0.2730  C2=0.7270 | C1= 0.1885  C2= 0.7265  C3= 0.0850 | C1= 0.1521  C2= 0.4963  C3=0.0815  C4= 0.2701 | C1=0.1741  C2=0.3539  C3= 0.1686  C4= 0.1664  C5= 0.1371 | C1=0.2024  C2= 0.0879  C3= 0.1921  C4= 0.1893  C5=0.1355  C6= 0.1927 | C1= 0.0748  C2=0.3111  C3=0.0601  C4= 0.2017  C5= 0.1369  C6=0.1103  C7=0.1049 | C1= 0.1194  C2=0.2891  C3=0.0613  C4= 0.0990  C5=0.1370  C6=0.0729  C7=0.1198  C8= 0.1017 |

**Supplementary data Table 4: Summary of Latent Class Diagnostics for Question 3**

(ᵻ) Lowest Information Criteria (IC) value
