# Supplementary material for: Public priorities for osteoporosis and fracture research: results from a general population survey
Source: Arch Osteoporos. 2017 Apr 28;12(1):45. doi: 10.1007/s11657-017-0340-5 (PMC5409917; doi:10.1007/s11657-017-0340-5)
Supplement: Supplementary file 6 — (DOCX 17 kb) [file 11657_2017_340_MOESM6_ESM.docx]

| ***1 Class*** |  | ***2 Classes*** | ***3 Classes*** | ***4 Classes*** | ***5 Classes*** | ***6 Classes*** | ***7 Classes*** | ***8 Classes*** |
| --- | --- | --- | --- | --- | --- | --- | --- | --- |
| 3089.24  3140.04  3108.28  3150.04  1.00 | *AIC*  *BIC*  *Sample Adj. BIC*  *Consistent AIC*  *Entropy*  *Bootstrapped*  *Likelihood Ratio*  *Test (SAS)* | 2939.65  3046.33  2979.63  3067.33  0.98  2 v 1  0.01 | 2828.73  2991.29  2889.65  3023.29 ᵻ  0.95  3 v 2  0.01 | 2770.48  2988.92  2852.34  3031.92  0.93  4 v 3  0.01 | 2798.16  3072.48  2900.96  3126.48  0.89  5 v 4  1 | 2705.61  3035.81  2829.34  3100.81  0.95  6 v 5 | 2681.78  3067.86  2826.45  3143.86  0.94  7 v 6 | 2522.72 ᵻ  2964.68 ᵻ  2688.34 ᵻ  3051.68  0.94  8 v 7 |
| C= | *% for each class* | C1= 0.3493  C2= 0.6507 | C1= 0.3491  C2= 0.3746  C3= 0.2763 | C1=0.0920  C2=0.5489  C3= 0.0396  C4= 0.3194 | C1= 0.0517  C2= 0.4558  C3= 0.0186  C4= 0.3147  C5=0.1593 | C1= 0.1569  C2= 0.3817  C3=0.0179  C4= 0.2710  C5= 0.0752  C6=0.0973 | C1= 0.1496  C2=0.2286  C3=0.0171  C4= 0.3994  C5=0.0427  C6= 0.0934  C7=0.0692 | C1= 0.0918  C2= 0.1611  C3= 0.0124  C4=0.0772  C5= 0.0454  C6=0.1145  C7=0.1245  C8=0.3732 |

**Supplementary data Table 5: Summary of Latent Class Diagnostics for Question 4**

(ᵻ) Lowest Information Criteria (IC) value
